# Crosstalk between Virulence Loci: Regulation of *Salmonella enterica* Pathogenicity Island 1 (SPI-1) by Products of the *std* Fimbrial Operon

**DOI:** 10.1371/journal.pone.0030499

**Published:** 2012-01-23

**Authors:** Javier López-Garrido, Josep Casadesús

**Affiliations:** Departamento de Genética, Universidad de Sevilla, Sevilla, Spain; University of Birmingham, United Kingdom

## Abstract

Invasion of intestinal epithelial cells is a critical step in *Salmonella* infection and requires the expression of genes located in *Salmonella* pathogenicity island 1 (SPI-1). A key factor for SPI-1 expression is DNA adenine (Dam) methylation, which activates synthesis of the SPI-1 transcriptional activator HilD. Dam-dependent regulation of *hilD* is postranscriptional (and therefore indirect), indicating the involvement of unknown cell functions under Dam methylation control. A genetic screen has identified the *std* fimbrial operon as the missing link between Dam methylation and SPI-1. We show that all genes in the *std* operon are part of a single transcriptional unit, and describe three previously uncharacterized ORFs (renamed *stdD*, *stdE*, and *stdF*). We present evidence that two such loci (*stdE* and *stdF*) are involved in Dam-dependent control of *Salmonella* SPI-1: in a Dam^−^ background, deletion of *stdE* or *stdF* suppresses SPI-1 repression; in a Dam^+^ background, constitutive expression of StdE and/or StdF represses SPI-1. Repression of SPI-1 by products of *std* operon explains the invasion defect of *Salmonella* Dam^−^ mutants, which constitutively express the *std* operon. Dam-dependent repression of *std* in the ileum may be required to permit invasion, as indicated by two observations: constitutive expression of StdE and StdF reduces invasion of epithelial cells *in vitro* (1,000 fold) and attenuates *Salmonella* virulence in the mouse model (>60 fold). In turn, crosstalk between *std* and SPI-1 may play a role in intestinal infections by preventing expression of SPI-1 in the caecum, an intestinal compartment in which the *std* operon is known to be expressed.

## Introduction

*Salmonella enterica* is a Gram-negative bacterium that causes intestinal and systemic diseases in a variety of animal hosts [1]. *Salmonella* is a typical foodborne pathogen, and infection usually starts by the ingestion of contaminated food or water [2]. *Salmonella* has the ability to penetrate epithelial cells in the small intestine, a process known as invasion [3], [4]. After invasion, the infection can remain localized in the intestine, producing gastroenteritis. In specific serovar-host combinations, however, *Salmonella* can cross the intestinal epithelial barrier and disseminate inside the host, producing a systemic, life-threatening infection (e. g., typhoid fever in humans). It has been estimated that >90 million cases of *Salmonella*-associated gastroenteritis and >20 million cases of typhoid fever occur per year worldwide, resulting in 155,000 and 200,000 deaths respectively [5], [6].

*Salmonella* and *Escherichia* are close relatives, and it has been estimated that genus divergence occurred 120–160 million years ago [7]. *Salmonella* pathogenicity has evolved by sequential acquisition of genetic elements, each contributing to distinct aspects of virulence [8], [9]. Amongst those elements are the *Salmonella* pathogenicity islands (SPIs), which are clusters of virulence genes located in the chromosome. More than 10 SPIs have been described [10], although some of them are serotype-specific. These regions are absent in the chromosome of other enterics and usually have a G+C content different from that of the *Salmonella* chromosome, suggesting that they have been acquired by horizontal transfer [9], [11]. This view is supported by two additional lines of evidence. First, SPIs are frequently inserted at tRNA genes [10], which are hotspots for the integration of foreign DNA elements [12], [13]. Second, some SPIs contain putative homologs of genes encoding integrases or transposases, and are flanked by direct repeats [8], [10].

**Figure 1:**
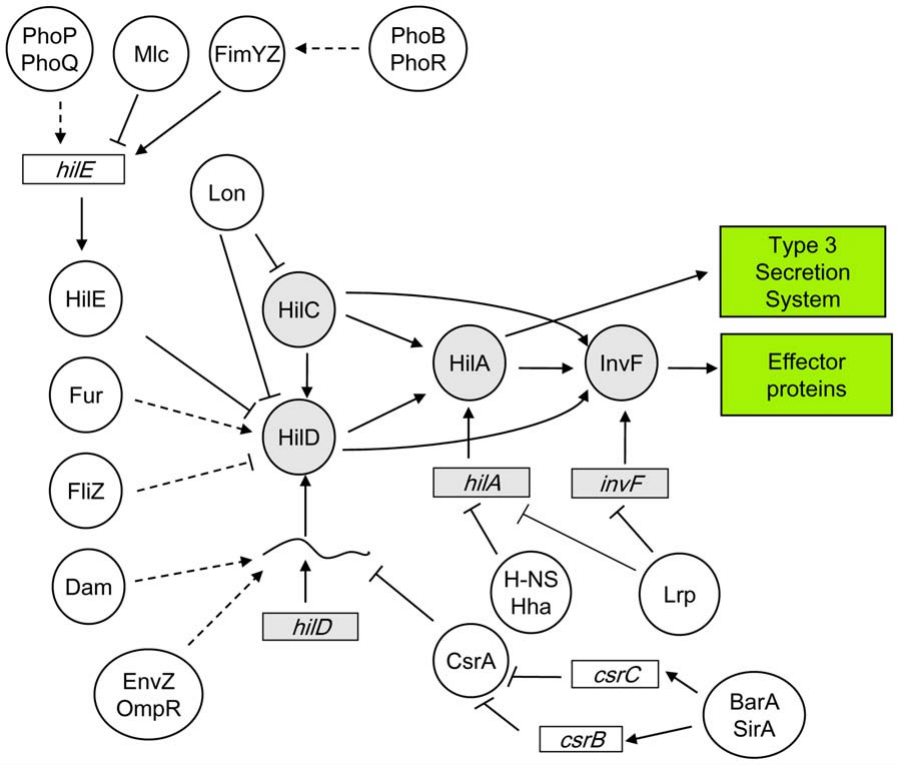
Diagram of SPI-1 regulation. Boxes represent genes, and circles represent proteins. Grey boxes and grey circles indicate SPI-1-encoded regulators. White circles indicate regulators encoded outside SPI-1.

*Salmonella* infection requires coordinated expression of virulence genes. During evolution, SPIs have been integrated into pre-existing regulatory networks, thus resulting in crosstalk between the *Salmonella* core genome and horizontally-acquired genetic elements [8]. One of the best characterized SPIs is the *Salmonella* pathogenicity island 1 (SPI-1), which is necessary for invasion of epithelial cells in the animal intestine. SPI-1 encodes a type 3 secretion system (TTSS) as well as effector proteins that are translocated into the eukaryotic cell cytoplasm [14]–[16]. SPI-1 expression is controlled by four SPI-1-encoded transcriptional activators: HilA, HilC, HilD, and InvF [14], [17]–[19]. These regulators form a regulatory network that incorporates regulatory inputs from global regulators. For instance, the leucine-responsive regulatory protein, Lrp, reduces SPI-1 expression by repressing transcription of *hilA* and *invF* [20]. The nucleoid-associated proteins H-NS and Hha repress *hilA* expression by direct binding to regions located upstream and downstream the *hilA* promoter [21], [22]. HilC and HilD are substrates for the ATP-dependent Lon protease [23], which contributes to SPI-1 repression after invasion of epithelial cells [24]. HilE is a negative regulator of SPI-1 [25] and may interfere with HilD function by direct protein-protein interaction [26]. Transcription of *hilE* is activated by the fimbrial regulator FimYZ [27], and is repressed by the PTS-dependent regulator Mlc [28], thus transmitting inputs to SPI-1 through HilD. In addition, the two-component systems PhoP/PhoQ and PhoB/PhoR may activate *hilE* expression [18], [19]. SP1-1 is also regulated by the Csr system [29]. Overexpression of *csrA* represses SPI-1 expression [29], [30]. CsrA binds to a region in *hilD* mRNA that overlaps with the ribosome-binding sequence, likely preventing translation and accelerating mRNA decay [30]. The two-component regulatory system BarA/SirA activates SPI-1 expression through the Csr pathway, activating transcription of the *csrB* and *csrC* genes, which encode CsrA antagonists [31]. Another activator of SPI-1 is the ferric uptake regulator, Fur, and the mechanism of regulation is controversial [32]–[34]. The EnvZ/OmpR two-component system activates SPI-1, likely by controlling *hilD* expression at the postranscriptional level [18], [35]. Furthermore, a recent report shows that FliZ, an RpoS inhibitor [36], activates SPI-1 expression by controlling HilD activity [37]. A diagram that summarizes SPI-1 regulation is shown in **Figure 1**.

In previous studies, we showed that DNA adenine (Dam) methylation is necessary to sustain a high level of SPI-1 expression [38]–[40]. Genetic analysis indicated that Dam-dependent regulation of SPI-1 is transmitted via HilD [39]. However, Dam-dependent regulation of *hilD* is not transcriptional but postranscriptional [39], and several lines of evidence suggest that a postranscriptional regulator whose synthesis is Dam-dependent may control *hilD* mRNA stability [39]. In this study, we show that Dam-dependent postranscriptional regulation of *hilD* is exerted by products encoded on another horizontally-acquired genetic element, the *std* fimbrial cluster. Std fimbriae belong to the chaperone-usher-dependent assembly class [41], [42]. StdA constitutes the major fimbrial subunit [43], [44], while StdB and StdC are a putative outer membrane usher protein and a putative periplasmic chaperone, respectively [45]. We have characterized 3 additional genes of unknown function in the *std* cluster, and have renamed them *stdD*, *stdE*, and *stdF*. Products of two such genes, *stdE* and *stdF*, turn out to be the molecular link between Dam methylation and SPI-1: lack of Dam methylation permits expression of the *std* operon, and the StdE and StdF products directly or indirectly downregulate *hilD* mRNA. These findings contribute to understand the invasion defect of *Salmonella* Dam^−^ mutants [40], [46] and the relief of virulence attenuation observed in Dam^−^ Std^−^ mutants [47]. Furthermore, we show that constitutive expression of StdE and StdF reduces invasion of epithelial cells and impairs *S. enterica* virulence in the mouse model. StdEF-mediated repression of SPI-1 may be an example of coordination in the control of virulence genes, preventing synthesis of the invasion machinery in enviroments which are not appropriate for invasion. One such compartment may be the caecum, an intestinal section in which Std fimbriae are known to be produced [48], [49].

## Materials and Methods

### Bacterial strains, bacteriophages, and standard strain construction

All the *Salmonella enterica* strains listed in **Table S1** belong to serovar Typhimurium, and derive from the mouse virulent strain ATCC 14028. For simplicity, *Salmonella enterica* serovar Typhimurium is often abbreviated as *S. enterica*. Targeted gene disruption was achieved using pKD4 or pKD13 [50]. Antibiotic resistance cassettes introduced during strain construction were excised by recombination with plasmid pCP20 [50]. The oligonucleotides used for disruption (labeled “UP” and “DO”) are listed in **Table S2**, together with the oligonucleotides (labeled “E”) used for allele verification by the polymerase chain reaction. For the construction of transcriptional and translational *lac* fusions in the *Salmonella* chromosome, FRT sites generated by excision of Km^r^ cassettes [50] were used to integrate either pCE37 or pCE40 [51]. Addition of a 3xFLAG epitope tag to protein-coding DNA sequences was carried out using plasmid pSUB11 (Km^r^, 3xFLAG) [52]. Transductional crosses using phage P22 HT 105/1 *int201* ([53] and G. Roberts, unpublished) were used for strain construction operations involving chromosomal markers. The transduction protocol was described elsewhere [54]. To obtain phage-free isolates, transductants were purified by streaking on green plates. Phage sensitivity was tested by cross-streaking with the clear-plaque mutant P22 H5.

### Growth conditions

Luria-Bertani (LB) broth was used as standard liquid medium. Solid media contained 1.5% agar. When needed, kanamycin sulfate (Km), chloramphenicol (Cm), or ampicillin (Ap) was added to LB at a final concentration of 50 µg/ml, 20 µg/ml and 100 µg/ml, respectively. Green plates were prepared according to Chan and co-workers [55], except that methyl blue (Sigma Chemical Co., St. Louis, MO) substituted for aniline blue. Plate tests for monitoring ß-galactosidase activity used 5-bromo-4-chloro-3-indolyl-ß-D-galactopyranoside (“X-gal”, Sigma Chemical Co.) as indicator. To monitor expression of SPI-1 genes by ß-galactosidase assays, Western blotting, or Northern blotting, saturated cultures were diluted 1∶50 in LB and incubated at 37°C with shaking (200 r. p. m.). Samples were taken when the cultures had reached the stationary phase (O.D._600_ = 2.0−2.5).

### Construction of a pBR328-based plasmid library of *Salmonella* genome

Genomic DNA from *Salmonella enterica* serovar Typhimurium ATCC 14028 was partially digested with Sau3A. DNA fragments 7–11 kb long were ligated to the pBR328 vector, previously digested with BamHI and dephosphorylated. *Salmonella* strain TR5878 was transformed with the ligation products, and ampicillin-resistant colonies were selected on LB+Ap plates. Pools of ∼1,000 independent transformants were collected and lysed with phage P22 HT 105/1 *int201*. As a quality control, we tested the ability of the library pools to complement null mutations in *araA* (required for growth with L-arabinose as the sole carbon source) or *xylA* (required for growth with D-xylose as the sole carbon source). Lysates that permitted sucessful complementation were stored and used for plasmid delivery to recipient strains in subsequent genetic screens.

### Construction of relevant strains

P_L*tetO*_ *-stdEF* and P_L*tetO*_ *-stdF* constructions were engineered by inserting the P_L*tetO*_ promoter [56] upstream *stdE* and *stdF* (respectively) on the *Salmonella* chromosome. In both constructions, P_L*tetO*_ insertion removed the upstream genes in the *std* operon and the native promoter upstream *stdA*. A fragment containing the *cat* gene and the P_L*tetO*_ promoter was amplified by PCR using pXG1 as template [57]. The primers were labelled P_L*tetO*_UP and P_L*tetO*_DO (**Table S2**). The PCR product was treated with DpnI to remove template traces. The construction was inserted in the chromosome by Lambda Red recombinase-mediated recombination [50], and Cm^r^ colonies were selected. Insertion of the construct was verified by PCR, using a pair of primers specific for the *cat* gene and the target gene (**Table S2**).

### Protein extracts and Western blot analysis

Total protein extracts were prepared from bacterial cultures grown at 37°C in LB until stationary phase (final O.D._600_∼2.5). Bacterial cells were collected by centrifugation (16,000 g, 2 min) and suspended in 100 µl of Laemmli sample buffer [1.3% SDS, 10% (v/v) glycerol, 50 mM Tris-HCl, 1.8% ß-mercaptoethanol, 0.02% bromophenol blue, pH 6.8]. Proteins were resolved by Tris-Tricine-PAGE (12%). Conditions for protein transfer have been described elsewhere [47]. Optimal dilutions of primary antibodies were as follows: anti-FLAG M2 monoclonal antibody (Sigma Chemical Co.), 1∶5,000; anti-GroEL polyclonal antibody (Sigma Chemical Co.), 1∶20,000. Goat anti-mouse horseradish peroxidase-conjugated antibody (1∶5,000, BioRad, Hercules, CA) or Goat anti-rabbit horseradish peroxidase conjugated antibody (1∶20,000, Santa Cruz Biotechnology, Heidelberg, Germany) were used as secondary antibodies. Proteins recognized by the antibodies were visualized by chemoluminescence using luciferin-luminol reagents in a LAS 3000 Mini Imaging System (Fujifilm, Tokyo, Japan). For quantification, the intensity of the bands was determined using MultiGauge software (Fujifilm). GroEL was used as loading control.

### Co-transcription analysis of *std* genes

RNA used for retrotranscription was extracted from *S. enterica* cultures grown in LB to stationary phase (O.D._600_∼2.5) using the SV total RNA isolation system (Promega Co., Madison, WI) as described at http://www.ifr.ac.uk/safety/microarrays/protocols.html. The quality of the preparation and the concentration of RNA were determined using a ND-1000 spectrophotometer (NanoDrop Technologies, Wilmington, DE). To avoid genomic DNA contamination, the preparation was treated twice with DNase I (Turbo DNA free, Applied Biosystems/Ambion, Austin, TX), following manufacturer's instructions. A 0.6 µg aliquot of DNase I-treated RNA was used for cDNA synthesis using the High Capacity cDNA Reverse Transcription Kit (Applied Biosystems, Foster City, CA). One µl of retrotranscribed cDNA was used as template for PCR with primer pairs specific for contiguous *std* ORFs (**Table S2**). Non-retrotranscribed RNA and genomic DNA were used as negative and positive controls, respectively.

### RNA extraction and Northern analysis

A 2 ml aliquot from a stationary culture (O.D._600_∼2) was centrifuged at 16,000 g, 4°C, during 5 min. The pellet was resuspended in 100 µl of a solution of lysozyme (Sigma Chemical Co.), 3 mg/ml. Cell lysis was facilitated by three consecutive freeze-thaw cycles. After lysis, RNA was extracted using 1 ml of Trizol reagent (Invitrogen Co, Carlsbad, CA), according to manufacter's instructions. Lastly, total RNA was resuspended in 30 µl of RNase-free water. The quality of the preparation and the RNA concentration were determined using a ND-1000 spectrophotometer (NanoDrop Technologies). For Northern blot analysis, 10 µg of total RNA was loaded per well and electrophoresed in denaturing 1% agarose formaldehyde gels. Vaccum transfer and fixation to Hybond-N^+^ membranes (GE Healthcare, Little Chalfont, UK) were performed using 0.05 M NaOH. Filters were then hybridized using an internally labelled [(^32^P)UTP] riboprobe specific for the upstream (5′) 300 nucleotides of the *hilD* coding sequence. Hybridization was carried out at 65°C. As a control of RNA loading and transfer efficiency, the filters were hybridized with a riboprobe for the RNase P mRNA gene (*rnpB*). Images of radioactive filters were obtained with a FLA-5100 imaging system (Fujifilm), and quantification was performed using MultiGauge software (Fujifilm).

### ß-galactosidase assays

Levels of ß-galactosidase activity were assayed using the CHCl_3_-sodium dodecyl sulfate permeabilization procedure [58]. ß-galactosidase activity data are the averages and standard deviations from ≥3 independent experiments. The Student's *t* test was used to determine if the differences in ß-galactosidase activities were statistically significant.

### Virulence assays in mice

Groups of 5 eight-weeks-old female BALB/c mice (Charles River Laboratories, Santa Perpetua de Mogoda, Spain) were inoculated with a 1∶1 ratio of two strains, one carrying the P_L*tetO*_ *-stdEF* construction and another carrying the P_L*tetO*_ *-stdEF ΔstdEF* construction. To differentiate between the two strains, a Mu*d*J transposon carrying a *lacZ* gene was inserted in the *trg* locus in the chromosome of the P_L*tetO*_ *-stdEF ΔstdEF* strain. The *trg::*Mu*d*J allele has been shown to be neutral for *Salmonella* virulence ([59]; F. Ramos-Morales, personal communication). For oral inoculation, bacterial cultures were grown overnight at 37°C in LB without shaking. Oral inoculation was performed by feeding the mice with 25 µl of saline containing 0.1% lactose and 10^8^ bacterial CFU. A competitive index (CI) for each mutant was calculated as the ratio between the mutant and the wild type strain in the output (bacteria recovered from the murine spleen after infection) divided by their ratio in the input (initial inoculum) [60], [61]. The Student's *t* test was used to determine whether the output ratio was or not significantly different from the input ratio.

### Ethics statement

Animal research adhered to the principles mandatory in the European Union, as established in the Legislative Act 86/609 CEE (November 24, 1986), and followed the specific protocols established by the Royal Decree 1201/2005 of the Government of Spain (October 10, 2005). The protocols employed in the study were reviewed by the Comité Ético de Experimentación de la Universidad de Sevilla, and were approved on January 16, 2010 (permit number 59-A-2010). All surgery was performed under sodium pentobarbital anesthesia, and all efforts were made to minimize suffering.

### Invasion assays

HeLa cells (ATCC CCL2) were cultured in tissue culture medium (Dulbecco's modified essential medium supplemented with 10% fetal calf serum and 2 mM L-glutamine). For routine cultivation, 60 µg/ml penicillin and 100 µg/ml streptomycin were added to the culture medium. The day before infection, approximately 1.5×10^5^ HeLa cells were seeded, using 24-well plates (Costar, Corning, New York, NY). Each well contained 1 ml of tissue culture medium without antibiotics. Cells were grown at 37°C, 5% CO_2_ to obtain 80% confluency. One hour before infection, the culture medium was removed and replaced by 0.5 ml fresh tissue culture medium without antibiotics. Bacteria were grown overnight at 37°C in LB with shaking, diluted into fresh medium (1∶50), and incubated at 37°C without shaking up to O.D._600_ 0.6–0.8 (overnight). Bacteria were added to reach a multiplicity of infection (MOI) of 50∶1 bacteria/HeLa cell. HeLa cells were infected for 30 min, washed 3 times with PBS, incubated in fresh tissue culture medium containing 100 µg/ml gentamicin for 1.5 hours, and washed 3 times with PBS. Numbers of viable intracellular bacteria were obtained by lysing infected cells with 1% Triton X-100 (prepared in PBS) and subsequent plating. Invasion rates were determined as the ratio between viable intracellular bacteria and viable bacteria added to infect the HeLa cells. Data are averages and standard deviations of 3 independent experiments. The Student's *t* test was used to determine the statistical significance of the differences observed.

## Results

### Genetic screen for regulators of *hilD* expression using a multicopy plasmid library of *Salmonella enterica*

We previously reported that regulation by Dam methylation is transmitted to SPI-1 via HilD [39]. However, Dam methylation was found to regulate *hilD* expression at the postranscriptional level, suggesting the involvement of additional, unknown regulators under Dam methylation control. The view that Dam-dependent regulation of *hilD* is indirect is further supported by the absence of GATC sites in the *hilD* promoter and upstream regulatory region, and by the observation that elimination of GATC sites in the *hilD* coding sequence does not abrogate Dam-dependent regulation (**Figure S1**). To search for Dam-dependent regulators of *hilD*, we considered two alternative possibilities: either Dam^+^ hosts might produce a factor that upregulates *hilD* or Dam^−^ hosts might produce a factor that downregulates *hilD*. We also considered the possibility that overexpression of the hypothetical regulator might render SPI-1 expression Dam-independent: namely, that overproduction of a repressor might downregulate SPI-1 in a Dam^+^ background, while overproduction of an activator might upregulate SPI-1 in Dam^−^ background. On these grounds, we devised a genetic screen for SPI-1 regulators in Dam^+^ and Dam^−^ backgrounds, using a pBR328-based multicopy plasmid library of the *Salmonella enterica* genome. As a reporter, we used a fusion (*hilD::lac930*) that bears the *lacZ* gene inserted immediately downstream the *hilD* stop codon and shows Dam-dependent expression (**Figure S2**).

Dam^+^ and Dam^−^ isogenic strains carrying the *hilD::lac930* fusion were transduced with 9 pools of the plasmid library, each containing around 1,000 independent clones. Transductants were selected on LB plates containing chloramphenicol and X-gal. Colonies with reduced ß-galactosidase activity (white) were sought in the Dam^+^ background, and colonies with increased ß-galactosidase activity (deep blue) were sought in the Dam^−^ background. Relevant results from these trials were as follows:

- Twelve independent candidates with increased ß-galactosidase activity were chosen amongst blue colonies obtained in the Dam^−^ screen. The fragments contained in the library plasmids were sequenced using specific primers flanking the insertion site (**Table S2**). DNA sequencing revealed that all pBR328 derivatives carried the same cloned fragment, which contained the *rtsA* gene amongst other genes (**Figure S3A**). Because RtsA is known to activate *hilD* transcription [35], we concluded that increased *hilD::lac930* expression was due to overproduction of RtsA. However, neither *rtsA* nor the other genes contained in the plasmid (*STM4310*, *STM4312*, *STM4313*, *STM4316*, *STM4317* and *STM4318*) are regulated by Dam methylation [38]. Hence, we ruled out that RtsA might be the Dam-dependent factor that controls *hilD* expression.
- In the Dam^+^ screen, five different plasmids were found to reduce *hilD::lac930* activity (**Figure S3B**). One such plasmid contained the *std* fimbrial gene cluster, amongst other neighboring genes (**Figure S3B**). The latter finding, together with transcriptomic data indicating that *std* mRNA is >100-fold more abundant in a Dam^−^ background [38], suggested that the *std* gene cluster might encode regulator(s) involved in SPI-1 repression in Dam^−^ mutants. None of the other loci present in the plasmids that reduced *hilD::lac930* activity (**Figure S3B**) is known to be under Dam methylation control [38]. Hence, further work was centered upon *std*.

### All genes in the *std* gene cluster are overexpressed in Dam^−^ mutants

**Figure 2:**
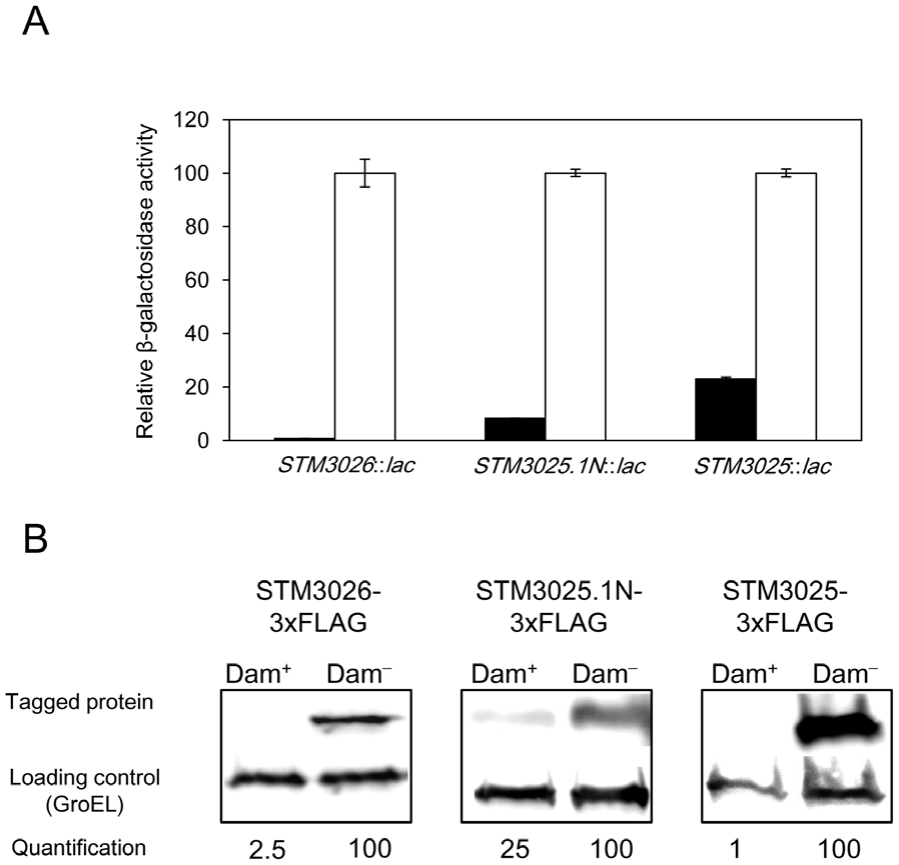
Expression of *STM3026* (*stdD*), *STM3025.1N* (*stdE*), and *STM3025* (*stdF*) in Dam^+^ and Dam^−^ backgrounds. A. ß-galactosidase activity of *STM3026*::*lac*, *STM3025.1N*::*lac*, and *STM3025*::*lac* translational fusions in a Dam^+^ background (black histograms) and in a Dam^−^ background (white histograms). ß-galactosidase activity has been relativized to 100 in the Dam^−^ background. Student's *t* test indicates that the differences observed in the ß-galactosidase activities of the fusions in a Dam^+^ and Dam^−^ backgrounds are statistically significant (P<0.005 in every case). B. Levels of STM3026 (StdD), STM3025.1N (StdE), and STM3025 (StdF) proteins in extracts from Dam^+^ and Dam^−^ hosts. 3xFLAG-tagged proteins were detected by Western blotting using anti-FLAG antibodies. GroEL was used as loading control. For quantification, the ratio tagged protein/GroEL was relativized to 100 in the Dam^−^ background.

Transcriptomic analysis had shown that *stdA*, *stdB*, *stdC*, and the uncharacterized putative genes *STM3026* and *STM3025* are all repressed by Dam methylation. Dam-dependent expression of *stdA*, *stdB*, and *stdC* had been confirmed by independent methods [38], [47]. However, the effect of Dam methylation on *STM3026* and *STM3025* expression had not been further analyzed. On the other hand, DNA sequence analysis *in silico* had indicated the existence of a sixth, uncharacterized open reading frame (ORF), designed *STM3025.1N*, in the intergenic region between *STM3026* and *STM3025* [45]. To update and complete previous data on Dam-dependent control of *std*, we compared the expression of *STM3026*, *STM3025.1N*, and *STM3025* in Dam^+^ and Dam^−^ backgrounds by two independent methods: (i) analysis of ß-galactosidase activity using *STM3026::lac*, *STM3025.1N::lac*, and *STM3025::lac* translational fusions (**Figure 2A**); and (ii) determination of STM3026, STM3025.1N, and STM3025 protein levels in protein extracts from Dam^+^ and Dam^−^ hosts, using protein variants tagged with the 3xFLAG epitope (**Figure 2B**). ß-galactosidase assays and Western blot analyses show that *STM3026*, *STM3025.1N*, and *STM3025* are expressed in a Dam^−^ background. Detection of the proteins by Western blot indicates that the three ORFs encode proteins indeed. Detection of Std proteins in a Dam^−^ background is consistent with previous observations indicating that the *std* operon is repressed under laboratory conditions and becomes derepressed in Dam^−^ mutants [38], [47]. To simplify and rationalize gene designations, *STM3026*, *STM3025.1N*, and *STM3025* have been renamed *stdD*, *stdE*, and *stdF*, respectively.

### *stdA*, *stdB*, *stdC*, *stdD*, *stdE*, and *stdF* constitute a polycistronic transcriptional unit

**Figure 3:**
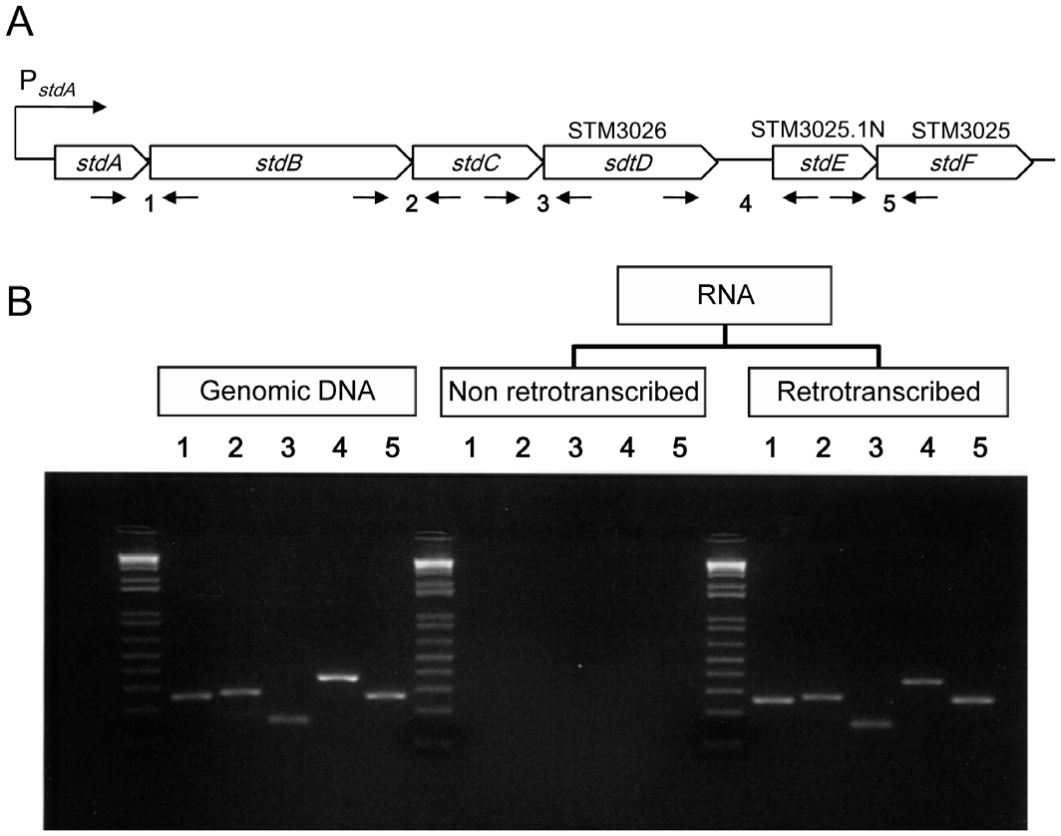
Evidence that *stdA*, *stdB*, *stdC*, *stdD*, *stdE*, and *stdF* constitute a polycistronic transcriptional unit. A. Diagram of the *std* operon. Opposite arrows below the diagram represent primer pairs used to examine co-transcription of contiguous coding sequences. B. Co-transcription of contiguous coding sequences in the *std* operon. PCR fragments generated with primer pairs specific for contiguous coding sequences were resolved in a 2% agarose gel, stained with ethidium bromide, and visualized with UV light. The 1 kb^+^ DNA ladder (Innovaplex, Sugar Land, TX) was used as size marker.

Expression of *stdA* is known to be driven by a promoter whose transcription is Dam-dependent [47]. The fact that expression of all the genes in the *std* cluster is upregulated in a Dam^−^ background suggested that the entire cluster might constitute a polycistronic unit transcribed from the *stdA* promoter. This hypothesis received experimental support from co-transcription analysis of contiguous ORFs by retrotranscription and PCR amplification. For this purpose, total RNA was extracted from a Dam^−^ mutant, and traces of DNA were removed by treatment with DNase I. The RNA sample was split in two fractions, one of which was retrotranscribed to cDNA using random primers; the other fraction underwent the same treatment but water was added instead of retrotranscriptase. Next, we performed PCR amplification with primer pairs specific for contiguous ORFs (**Figure 3A**, **Table S2**) in the presence of the following templates: *Salmonella* genomic DNA as positive control, nonretrotranscribed RNA as negative control, and cDNA as the experimental query. The PCR products were resolved in a 2% agarose gel with 0.5 µg/ml ethidium bromide, and visualized under UV light. As shown in **Figure 3B**, PCR products of the expected sizes were obtained using either genomic DNA or cDNA. However, no fragment was observed when RNA was used as template. These results indicate that the six genes in the *std* cluster constitute a polycistronic operon, transcribed from the promoter previously identified upstream *stdA* ([47]; **Figure 3A**). Our results, however, do not rule out the possibility that internal promoters may also exist.

### The *stdE* and *stdF* gene products are functional links between Dam methylation and SPI-1

**Figure 4:**
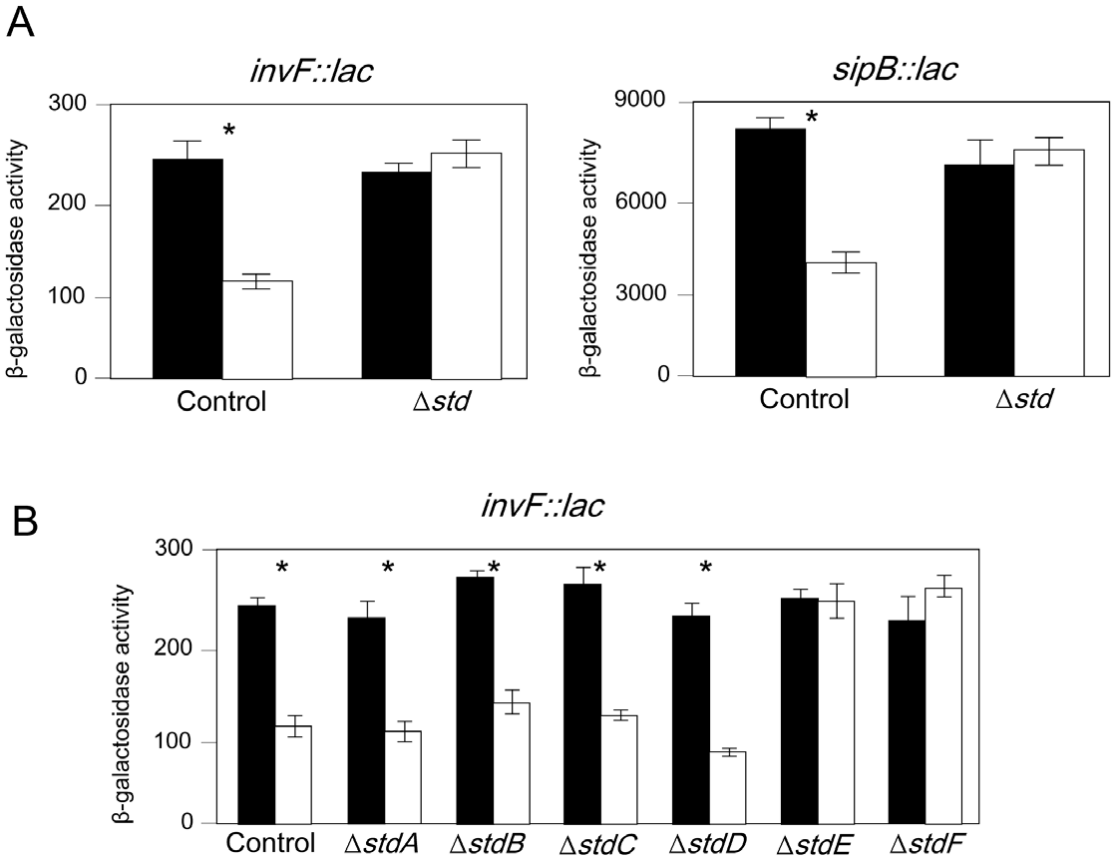
Identification of *std* operon products involved in SPI-1 repression. A. ß-galactosidase activities of *invF::lac* and *sipB::lac* fusions in a strain containing an intact *std* operon (control) and in a isogenic strain lacking the entire *std* operon (Δ*std*). Black and white histograms represent ß-galactosidase activities in Dam^+^ and Dam^−^ backgrounds, respectively. Asterisks above columns indicate that the differences observed are statistically significant (P<0.005). B. Regulation of an *invF::lac* fusion by Dam methylation in strains carrying in-frame deletions in individual *std* genes. Histograms represent ß-galactosidase activities in a Dam^+^ background (black) and in a Dam^−^ background (white). Asterisks above columns indicate that the differences observed are statistically significant (P<0.005).

If overexpression of the *std* operon was the cause of SPI-1 repression in Dam^−^ mutants (**Figure S3B**), we reasoned, SPI-1 repression in a Dam^−^ background should be suppressed by deletion of the *std* operon. On these grounds, we compared the expression of *invF::lac* and *sipB::lac* fusions in isogenic Dam^+^ and Dam^−^ strains that contained either an intact *std* operon or a complete deletion of *std*. As shown in **Figure 4A**, the ß-galactosidase activities of the *invF::lac* and *sipB::lac* fusions were reduced in a Dam^−^ background in the presence of a functional *std* operon. However, in a strain lacking the *std* operon, both fusions displayed similar ß-galactosidase activities in Dam^+^ and Dam^−^ backgrounds. These results support the hypothesis that one or more proteins encoded in the *std* operon are involved in the transmission of Dam-dependent regulation to SPI-1. In an attempt to identify such protein(s), Dam-dependent regulation of an *invF::lac* fusion was monitored in a set of mutants carrying in-frame, non-polar deletions in individual *std* genes. **Figure 4B** shows that *invF::lac* expression remains Dam-dependent in strains lacking *stdA*, *stdB*, *stdC*, and *stdD*, suggesting that these genes are not required for Dam-dependent control of SPI-1. However, repression of *invF::lac* in a Dam^−^ background is suppressed in strains lacking either *stdE* or *stdF*, suggesting that the products of both genes are necessary for SPI-1 repression in Dam^−^ mutants.

### StdE and StdF independently repress SPI-1 expression

**Figure 5:**
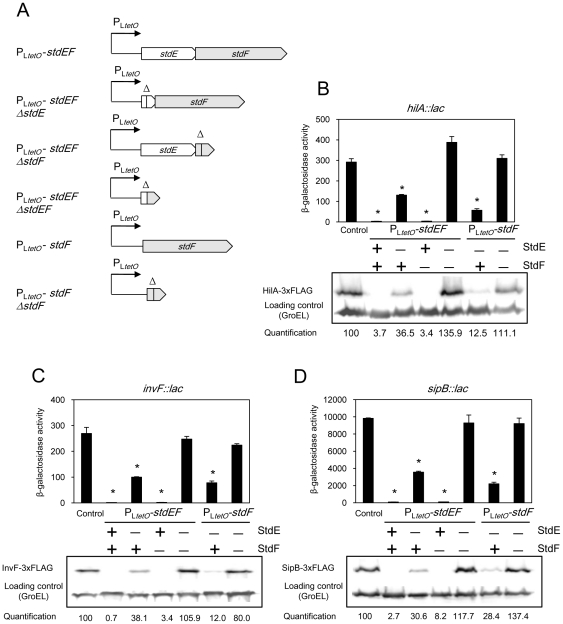
Downregulation of SPI-1 by StdE and StdF. A. Diagram representing P_L*tetO*_ *-stdEF* and P_L*tetO*_ *-stdF* constructions, and their respective deletion controls lacking *stdE*, *stdF*, or both. B–D. Expression of *hilA*, *invF*, and *sipB* in strains carrying a native *std* operon (control), P_L*tetO*_ *-stdEF*, P_L*tetO*_ *-stdF*, and their respective control constructs. Histograms represent ß-galactosidase activities of *hilA::lac*, *invF::lac*, and *sipB::lac* fusions. An asterisk indicates a statistically significant difference (P<0.005) compared to the control. HilA-3XFLAG, InvF-3xFLAG, and SipB-3xFLAG levels were determined by Western blotting using anti-FLAG antibodies. GroEL was used as loading control. For quantification, the ratio tagged protein/GroEL was relativized to 100 in control samples. The symbols “+” and “−”indicate the presence or the absence of StdE or StdF.

The above experiments provide a tentative explanation for the SPI-1 expression defect observed in the absence of Dam methylation [38], [39]: in a Dam^−^ background, the *std* operon is expressed, and the *stdE* and *stdF* gene products repress SPI-1. If this model was correct, we reasoned, constitutive expression of *stdE* and *stdF* should repress SPI-1 expression in a Dam^+^ background. To test this hypothesis, we placed *stdE* and *stdF* under the control of the P_L*tetO*_ promoter [56] to achieve moderate, Dam-independent expression of *stdE* and *stdF*. To avoid potential artefacts (e. g., caused by autogenous regulation of the *std* operon), the native *stdA* promoter and all the *std* genes upstream *stdE* were deleted. Two basic constructions were made: (i) P_L*tetO*_ *-stdEF* in which P_L*tetO*_ was placed upstream *stdE* on the chromosome, thus permitting constitutive expression of both *stdE* and *stdF*; (ii) P_L*tetO*_ *-stdF*, in which P_L*tetO*_ was inserted right upstream *stdF*, thus expressing *stdF* only. As controls, we used derivatives of the same strains which carry in-frame deletions in *stdE*, *stdF*, or in both genes (**Figure 5A**). Production of StdE and StdF in strains carrying the P_L*tetO*_ *-stdEF* or P_L*tetO*_ *-stdF* constructions was monitored by Western blotting, using protein variants tagged with the 3xFLAG epitope (**Figure S4**).

The effect of constitutive expression of *stdE* and *stdF* on the expression of SPI-1 genes *hilA*, *invF*, and *sipB* was monitored in strains carrying P_L*tetO*_-*stdEF*, P_L*tetO*_-*stdF* and the appropriate deletion controls. Two independent methods were used: (i) ß-galactosidase activities were measured in strains carrying *hilA::lac*, *invF::lac*, and *sipB::lac* fusions; (ii) HilA, InvF, and SipB protein levels were monitored by Western blotting, using protein variants tagged with the 3xFLAG epitope. The results obtained with both methods were congruent (**Figure 5** **, panels B–D**), and can be summarized as follows:

- Expression of *hilA*, *invF*, and *sipB* is strongly downregulated when P_L*tetO*_ is inserted upstream *stdE*.
- Downregulation is partially relieved when *stdE* is deleted. However, deletion of *stdF* alone does not restore SPI-1 expression. Deletion of both genes completely restores SPI-1 expression to wild type level, suggesting that SPI-1 downregulation is due to expression of both *stdE* and *stdF*.
- Insertion of P_L*tetO*_ upstream *stdF* downregulates the expression of *hilA*, *invF*, and *sipB*, but less efficiently than P_L*tetO*_ insertion upstream *stdE*.
- Deletion of *stdF* completely suppresses SPI-1 downregulation.

Altogether, these observations provide evidence that both StdE and StdF downregulate SPI-1 expression. In addition, both gene products downregulate SPI-1 independently: each is able to downregulate SPI-1 in the absence of the other.

### StdE and StdF regulate *hilD* expression at the postranscriptional level

**Figure 6:**
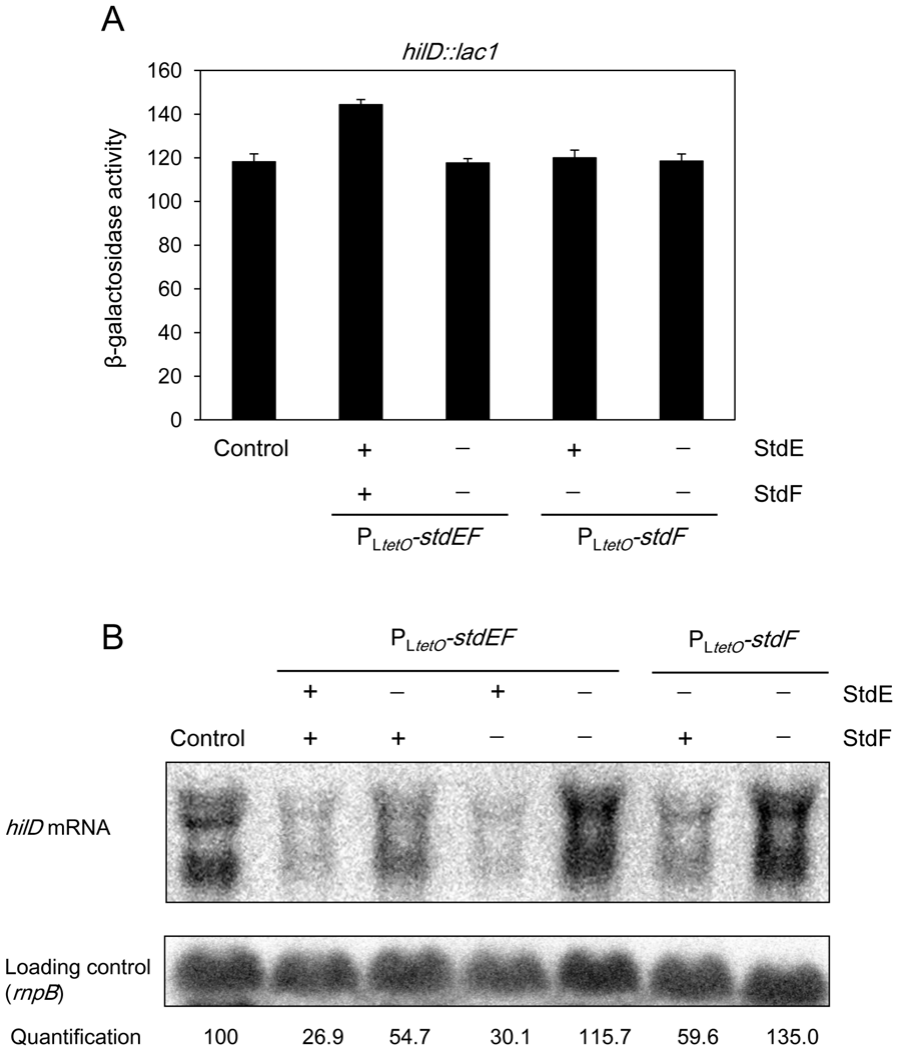
Postranscriptional regulation of *hilD* expression by StdE and StdF. A. ß-galactosidase activity of a *hilD::lac* fusion in a strain with a native *std* operon (control), and in strains carrying P_L*tetO*_ *-stdEF*, P_L*tetO*_ *-stdEF* Δ*stdEF*, P_L*tetO*_ *-stdF*, or P_L*tetO*_ *-stdF* Δ*stdF* constructions. The symbols “+” and “−”indicate the presence or the absence of StdE and StdF. None of the backgrounds shows ß-galactosidase activity significantly lower than the control. B. Level of *hilD* mRNA in RNA extracts from the wild type, P_L*tetO*_ *-stdEF*, P_L*tetO*_ *-stdF*, and their respective control strains. Levels of *hilD* mRNA were monitored by Northern blotting, using a riboprobe specific for the first (5′) 300 nucleotides of the *hilD* coding sequence. The symbols “+” and “−”indicate the presence or the absence of StdE and StdF.

We previously reported that regulation of *hilD* by Dam methylation is postranscriptional [39]. After finding that Dam-dependent *hilD* regulation depended on StdE and StdF, it seemed logical to expect that StdE and StdF would repress *hilD* expression at the postranscriptional level. To test this hypothesis, we examined *hilD* expression using a transcriptional fusion (*hilD::lac1*) in which *lacZ* is inserted exactly at the *hilD* transcription start site. The *hilD::lac1* fusion faithfully reproduces transcriptional regulation of *hilD*, as indicated by the observation that the fusion is upregulated in the presence of a multicopy plasmid that expresses RtsA, a transcriptional activator of *hilD* [35] (**Figure S5**). The expression level of *hilD::lac1* was determined in the wild type as well as in the P_L*tetO*_ *-stdEF*, P_L*tetO*_ *-stdEF ΔstdEF*, P_L*tetO*_ *-stdF*, and P_L*tetO*_ *-stdF ΔstdF* backgrounds (**Figure 5A** **,** **Figure 6A**). The ß-galactosidase activities were similar in all strains, suggesting that StdE and StdF do not regulate *hilD* transcription initiation.

The possibility that StdE and StdF downregulate *hilD* expression at the postranscriptional level was examined by analyzing *hilD* mRNA levels by Northern blot in the following backgrounds: wild type, P_L*tetO*_ *-stdEF*, P_L*tetO*_ *-stdEF ΔstdE*, P_L*tetO*_ *-stdEF ΔstdF*, P_L*tetO*_ *-stdEF ΔstdEF*, P_L*tetO*_ *-stdF*, and P_L*tetO*_ *-stdF ΔstdF*. As shown in **Figure 6B**, the level of *hilD* mRNA is reduced around 4 fold in the P_L*tetO*_ *-stdEF* strain. Deletion of *stdE* partially restores the *hilD* mRNA level, and simultaneous deletion of *stdE* and *stdF* completely restores *hilD* mRNA to wild type level. Furthermore, the amount of *hilD* mRNA is reduced twofold in the P_L*tetO*_ *-stdF* background, and this reduction is completely abolished by an *stdF* deletion. Altogether, these observations support the view that StdE and StdF downregulate *hilD* expression at the postranscriptional level.

### Expression of *stdE* and *stdF* inhibits *Salmonella* invasion

**Figure 7:**
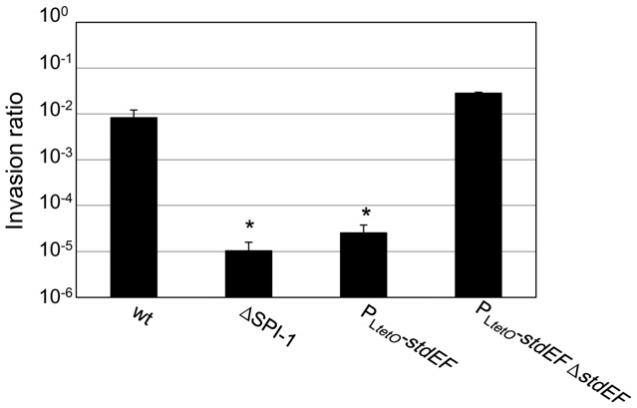
Effect of *stdE* and *stdF* expression on *Salmonella* invasion *in vitro*. The invasion ratios of epithelial HeLa cells by four *Salmonella* strains are represented: the wild type (wt), a strain with a deletion of SPI-1 (*ΔSPI-1*), a strain carrying the P_L*tetO*_ *-stdEF* construction, and a strain carrying the P_L*tetO*_ *-stdEF* Δ*stdEF* construction. Histograms represent averages and standard deviations of 3 independent experiments. An asterisk indicates a statistically significant difference (P<0.05) compared with the wild type.

Because constitutive expression of *stdE* and *stdF* represses SPI-1 (**Figure 5**; **Figure 6**) and SPI-1 is essential for *Salmonella* invasion, we examined the invasiveness of *S. enterica* ser. Typhimurium upon constitutive expression of *stdE* and *stdF*. For this purpose, we compared the ability of *Salmonella* strains carrying either the P_L*tetO*_ *-stdEF* or P_L*tetO*_ *-stdEF ΔstdEF* constructions to invade epithelial HeLa cells *in vitro*. As a positive control, we used the wild type strain. As a negative control, we used a strain with a deletion of SPI-1 *(ΔSPI-1)*. As shown in **Figure 7**, the *ΔSPI-1* strain is approximately 1,000 fold less invasive than the wild type. Constitutive expression of *stdE* and *stdF* (P_L*tetO*_ *-stdEF*) produces an invasion defect similar to deletion of SPI-1 (**Figure 7**), and this defect is suppressed by mutation of *stdE* and *stdF* (P_L*tetO*_ *-stdEF ΔstdEF*) (**Figure 7**). Hence, constitutive expression of *stdE* and *stdF* inhibits *Salmonella* invasion.

### Expression of *stdE* and *stdF* attenuates *Salmonella* virulence in mice

The observation that expression of *stdE* and *stdF* inhibits *Salmonella* invasion *in vitro* led us to speculate that StdE and StdF might also inhibit invasion of epithelial cells in the animal intestine. If such was the case, we reasoned, a *Salmonella* strain which constitutively expresses *stdE* and *stdF* should be attenuated in mice infected by the oral route. To test this hypothesis, we compared the virulence of *Salmonella* strains that expressed or not *stdE* and *stdF*. Five BALB/c mice were orally inoculated with a 1∶1 mixture of a strain that constitutively expressed *stdE* and *stdF* (P_L*tetO*_ *-stdEF*) and an isogenic strain carrying a deletion of both *stdE* and *stdF* (P_L*tetO*_ *-stdEF ΔstdEF*). To distinguish the two strains on plates, we inserted a Mu*d*J transposon at the *trg* chromosomal locus of the P_L*tetO*_ *-stdEF ΔstdEF* strain. The *trg::*Mu*d*J allele has been shown to be neutral for *Salmonella* virulence ([59]; F. Ramos-Morales, personal communication). The competitive index of P_L*tetO*_ *-stdEF* strain (expressing *stdE* and *stdF*) *versus* the P_L*tetO*_ *-stdEF ΔstdEF* strain (lacking *stdE* and *stdF*) was 0.016 (**Table 1**), indicating that constitutive expression of *stdE* and *stdF* attenuates *Salmonella* virulence by the oral route more than 60 fold.

**Table 1:** Effect of constitutive expression of *stdE* and *stdF* in oral competition assays.

	Strain number	Relevant genotype	CI (A/B)	P
Strain A	SV6503	P_L*tetO*_-*stdEF*	0.016	<0.0001
Strain B	SV6901	P_L*tetO*_-*stdEF* Δ*stdEF*		

## Discussion

Evolutionary acquisition of genetic modules has provided *Salmonella* with new abilities to interact with eukaryotic cells and to exploit a variety of niches [8], [11]. The *std* gene cluster is conserved amongst *Salmonella* serovars and absent in closely related species [11], suggesting horizontal acquisition. A critical requirement of modular evolution is to coordinate expression of the genetic modules, which in some cases carry regulatory genes that provide connections with the core genome [8]. In addition, examples of crosstalk between horizontally acquired modules have been described: HilD, a regulator encoded by SPI-1, activates SPI-2 expression during late stationary phase [62]; expression of SPI-4 genes is activated by SprB, a transcriptional regulator encoded on SPI-1 [63]; HilE, a SPI-1 negative regulator, is encoded on a region of *Salmonella* chromosome that has been proposed to be a pathogenicity island [26]; SPI-1 and SPI-2 transcriptional regulators control the synthesis of effector proteins encoded outside the islands [64], [65], often in horizontally-acquired DNA fragments [66], [67]. In this study, we describe a connection between *std* and SPI-1 which may be viewed as a novel example of crosstalk between horizontally-acquired, virulence-related genes.

We have characterized three putative ORFs in the *std* gene cluster, previously annotated as *STM3026*, *STM3025.1N*, and *STM3025*. Western blot analyses have confirmed the existence of three proteins, which have been renamed StdD (STM3026), StdE (STM3025.1N), and StdF (STM3025) respectively. *In silico* analysis indicates that StdE and StdF may be cytoplasmic proteins, while StdD may be an outer membrane protein.

Evidence that *stdA*, *stdB*, *stdC*, *stdD*, *stdE*, and *stdF* may constitute a polycistronic transcriptional unit is provided by simultaneous upregulation of all six genes in a Dam^−^ background (**Figure 2**; [38], [47]), and by retrotranscription and PCR amplification showing that the six genes are co-transcribed (**Figure 3**). Transcription of *std* is driven by a promoter located upstream *stdA* [47]. Transcription from P*_stdA_* is activated by binding of HdfR, a LysR-like factor, to a regulatory region upstream the promoter. However, methylation of two GATC sites in the regulatory region prevents binding of HdfR, thus repressing *std* expression ([47], Jakomin *et al.*, in preparation). Altogether, these observations suggest that all *std* genes may be coordinately regulated by Dam methylation upon transcription from P*_stdA_*. However, internal promoters may also exist.

Dam^−^ mutants of *Salmonella enterica* are attenuated in the mouse model and present a plethora of virulence-related defects both at the intestinal stage of the infection and during systemic infection [40], [46], [68]. A relevant defect during intestinal infection is reduced SPI-1 expression, which is a consequence of *hilD* mRNA instability in Dam^−^ mutants [38], [39]. Genetic screens and subsequent experiments described in this study have identified the *std* fimbrial operon as the missing link between Dam methylation and SPI-1: (i) a multicopy plasmid containing the *std* operon downregulates *hilD* expression (**Figure S3B**); (ii) *std* genes are upregulated in Dam^−^ background (**Figure 2**; [38], [47]); and (iii) SPI-1 regulation by Dam methylation is completely suppressed in a strain lacking the *std* operon (**Figure 4A**). Altogether, these results suggest that expression of *std* in Dam^−^ mutants leads to SPI-1 repression. Our observations may also explain the intriguing observation that the extreme attenuation of *S. enterica* Dam^−^ mutants upon oral infection [40], [46] is partially suppressed by deletion of *std* [47].

**Figure 8:**
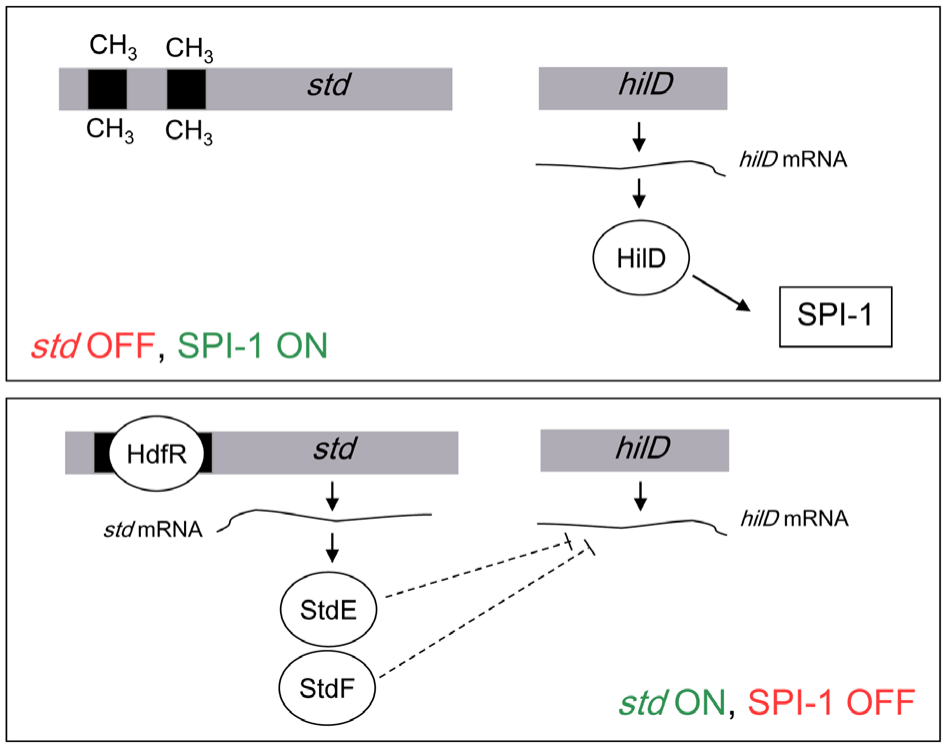
Model for crosstalk between the *std* operon and SPI-1. Lack of *std* expression (e. g., under laboratory conditions and in the ileum) permits HilD synthesis and subsequent expression of SPI-1. Transcription of *std* (e. g., in Dam^−^ mutants and in the caecum) yields StdE and StdF products, resulting in postranscriptional inhibition of HilD synthesis, and subsequent SPI-1 repression.

Epistasis analysis indicates that Dam-dependent control of SPI-1 requires both StdE and StdF. This conclusion is further supported by the following observations: (i) constitutive expression of *stdE* and *stdF* in a Dam^+^ background represses SPI-1 expression (**Figure 5**); (ii) StdE and StdF are overproduced in Dam^−^ background (**Figure 2**); (iii) Dam methylation, StdE, and StdF regulate SPI-1 expression through HilD (**Figure 6**; [39]); and (iv) as in the case of Dam methylation, StdE and StdF do not regulate *hilD* transcription but control the level of *hilD* mRNA (**Figure 6**). However, it remains to be established whether StdE and StdF directly control the *hilD* mRNA level or the regulatory mechanism involves intermediaries. A tentative model to explain SPI-1 regulation by Dam methylation is depicted in **Figure 8**: in a Dam^+^ background, GATC sites in the P*_stdA_* regulatory region are methylated, thus preventing binding of HdfR and subsequent activation of *std* transcription. In the absence of Dam methylation, HdfR activates transcription from P*_stdA_* and all the proteins encoded in the operon are produced. StdE and StdF then repress *hilD* expression at the postranscriptional level. As a consequence, the entire SPI-1 is downregulated.

A corollary from the above results is that *Salmonella* invasion can be expected to be inhibited whenever the *std* operon is expressed (**Figure 8**). This expectation was fulfilled by invasion assays *in vitro* showing that expression of StdE and StdF causes a >1,000 fold reduction in HeLa cell invasion (**Figure 7**). Furthermore, expression of StdE and StdF was found to attenuate *Salmonella* virulence about 60 fold upon infection of BALB/c mice (**Table 1**).

Under laboratory growth conditions, the *std* operon is tightly repressed [38], [43], [44], [47]. However, several observations indicate that Std fimbriae are produced in the animal intestine: (i) mice infected with serovar Typhimurium seroconvert to StdA, the major fimbrial component of Std fimbriae [43]; (ii) *std* deletion reduces the ability of *Salmonella* to colonize and persist in the caecum of infected mice, while it has no consequence for colonization of the small intestine [48]; and (iii) Std fimbriae bind α(1,2)fucose residues, which are abundant in the cecal mucosa [49]. In turn, *Salmonella* invasion takes place preferentially in the ileum [3] and is inhibited in the caecum [69]. Hence, it is tempting to speculate that StdE and StdF may play a role in SPI-1 expression inhibition in the caecum, an intestinal compartment which is not appropriate for invasion.

StdE shares 40–50% identity with the transcriptional activators GrlA and CaiF from *E. coli* and *Enterobacter cloacae*, respectively [70], [71]. Interestingly, StdF is related to an uncharacterized protein encoded immediately downstream CaiF in the *E. cloacae* chromosome [70], [71], which is part of a hypothetical fimbrial gene cluster whose genetic organization is reminiscent of the *std* operon [72]. StdF is also related to the SPI-1 *Salmonella* protein SprB, a transcriptional regulator that represses the *hilD* promoter and activates the *siiA* promoter [63]. Even though StdE and StdF are similar to known transcriptional regulators, they do not regulate *hilD* at the transcriptional level but at the postranscriptional level. Thus, these proteins may have either acquired the ability to control *Salmonella* gene expression at the postranscriptional level or may regulate transcription of a postranscriptional regulator of *hilD*. If the latter view is correct, crosstalk between *std* and SPI-1 may turn out to be more complex than described in this study, perhaps involving elements of the *Salmonella* core genome.

## Supporting Information

Figure S1 Elimination of the GATC sites in the *hilD* coding sequence does not alter the control of SPI-1 expression by Dam methylation. A. Diagram showing the distribution of GATCs within *hilD*; each CH_3_ represents a GATC. B. Site-directed mutagenesis of *hilD* GATCs. A single nucleotide exchange was introduced at each GATC site, generating a synonimous codon (shown in red). C. ß-galactosidase activity of an *invF::lac* fusion in Dam^+^ (black histograms) and Dam^−^ (white histograms) isogenic backgrounds. Measurements were performed in a strain that contained the 3 GATCs in the *hilD* coding sequence (control), and in a strain in which the 3 GATCs had been mutated (GATC*123). The differences observed between Dam^+^ and Dam^−^ are statistically significant in both strains (P<0.005).
(TIF)

Figure S2 ß-galactosidase activity of the *hilD::lac930* fusion in a Dam^+^ background (black histogram), and in a Dam^−^ background (white histogram). The differences observed are statistically significant (P<0.005).
(TIF)

Figure S3 ß-galactosidase activity of the *hilD::lac930* fusion in control strains (carrying pBR328), in candidates showing increased ß-galactosidase activity in a Dam^−^ background (A) and in candidates showing reduced ß-galactosidase activity in a Dam^+^ background (B). Diagrams representing the fragments harbored by the plasmids present in the candidates are also shown.
(TIF)

Figure S4 Production of StdE and StdF in strains carrying P_L*tetO*_ *-stdEF* and P_L*tetO*_ *-stdF* constructions A. Diagrams of strain construction and StdE and StdF tagging. B. Levels of StdE-3xFLAG and StdF-3xFLAG in protein extracts from the wild type, Dam^−^, P_L*tetO*_ *-stdEF*, and P_L*tetO*_ *-stdF* strains. 3xFLAG-tagged proteins were detected by Western blotting using a commercial anti-FLAG antibody. GroEL was used as loading control. For quantification, the ratio tagged protein/GroEL was relativized to 100 in the Dam^−^ background.
(TIF)

Figure S5 ß-galactosidase activity of the *hilD::lac1* fusion in a strain carrying pBR328 (black histogram), and in a strain carrying a pBR328 derivative that contains the *rtsA* gene (white histogram). The differences observed are statistically significant (P<0.005).
(TIF)

Table S1 Strain list.
(DOC)

Table S2 Oligonucleotides used in this study (5′→3′).
(DOC)
