# Supplementary figures and images for: Crosstalk between Virulence Loci: Regulation of Salmonella enterica Pathogenicity Island 1 (SPI-1) by Products of the std Fimbrial Operon
Source: PLoS One. 2012 Jan 23;7(1):e30499. doi: 10.1371/journal.pone.0030499 (PMC3264584; doi:10.1371/journal.pone.0030499)

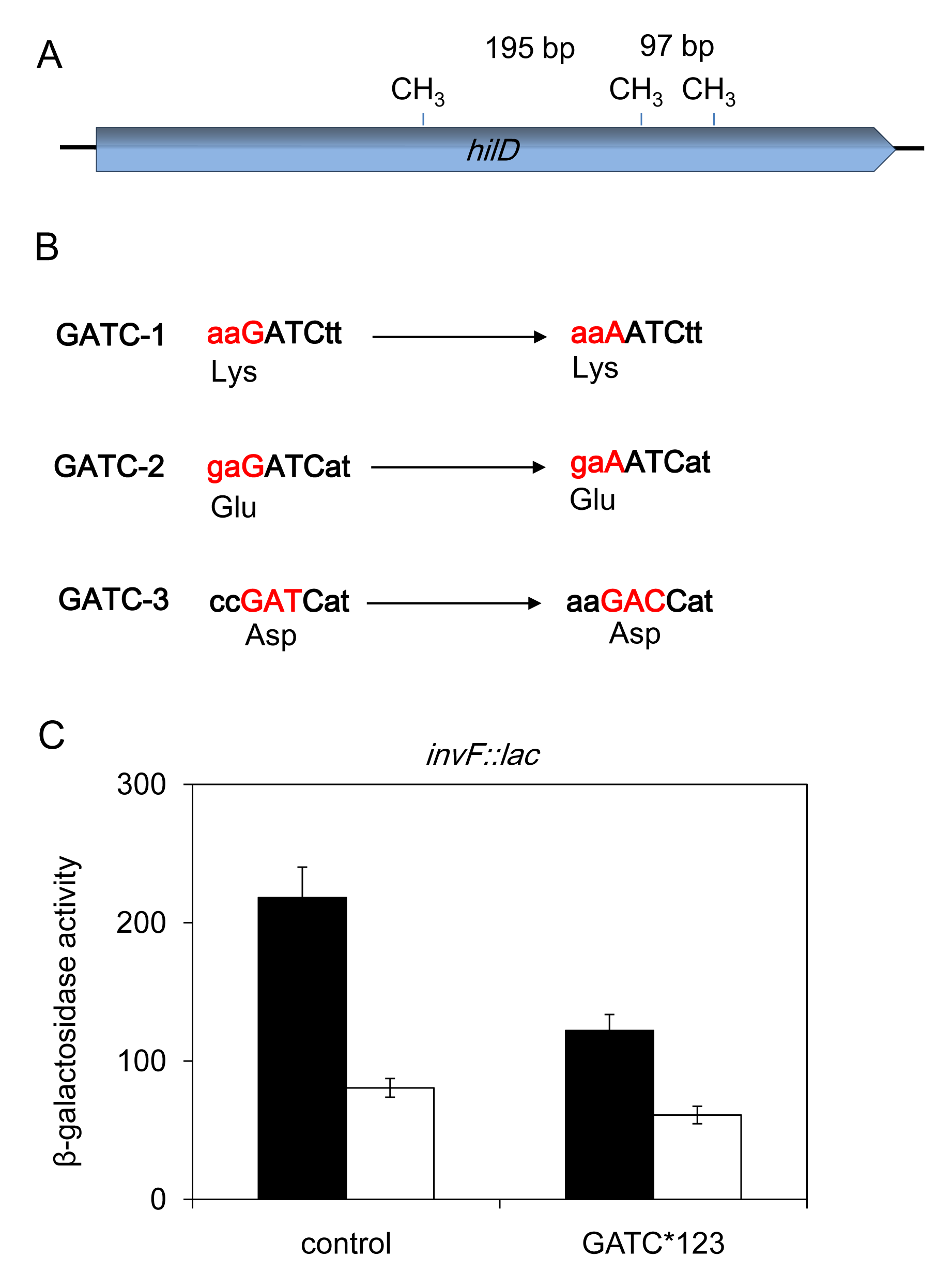

Supplement: Figure S1 — Elimination of the GATC sites in the hilD coding sequence does not alter the control of SPI-1 expression by Dam methylation. A. Diagram showing the distribution of GATCs within hilD; each CH3 represents a GATC. B. Site-directed mutagenesis of hilD GATCs. A single nucleotide exchange was introduced at each GATC site, generating a synonimous codon (shown in red). C. ß-galactosidase activity of an invF::lac fusion in Dam+ (black histograms) and Dam− (white histograms) isogenic backgrounds. Measurements were performed in a strain that contained the 3 GATCs in the hilD coding sequence (control), and in a strain in which the 3 GATCs had been mutated (GATC*123). The differences observed between Dam+ and Dam− are statistically significant in both strains (P<0.005). (TIF) [file pone.0030499.s001.tif]

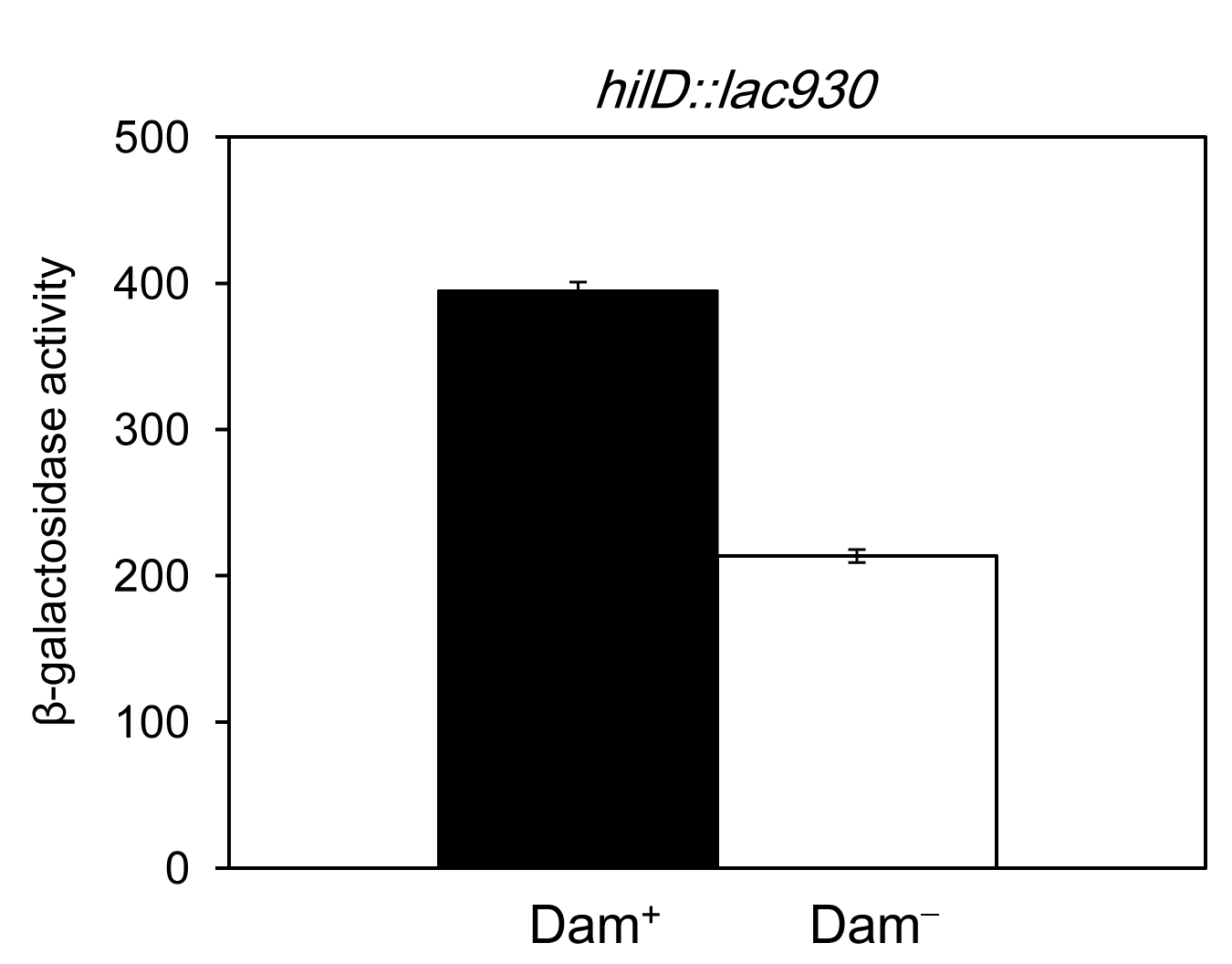

Supplement: Figure S2 — ß-galactosidase activity of the hilD::lac930 fusion in a Dam+ background (black histogram), and in a Dam− background (white histogram). The differences observed are statistically significant (P<0.005). (TIF) [file pone.0030499.s002.tif]

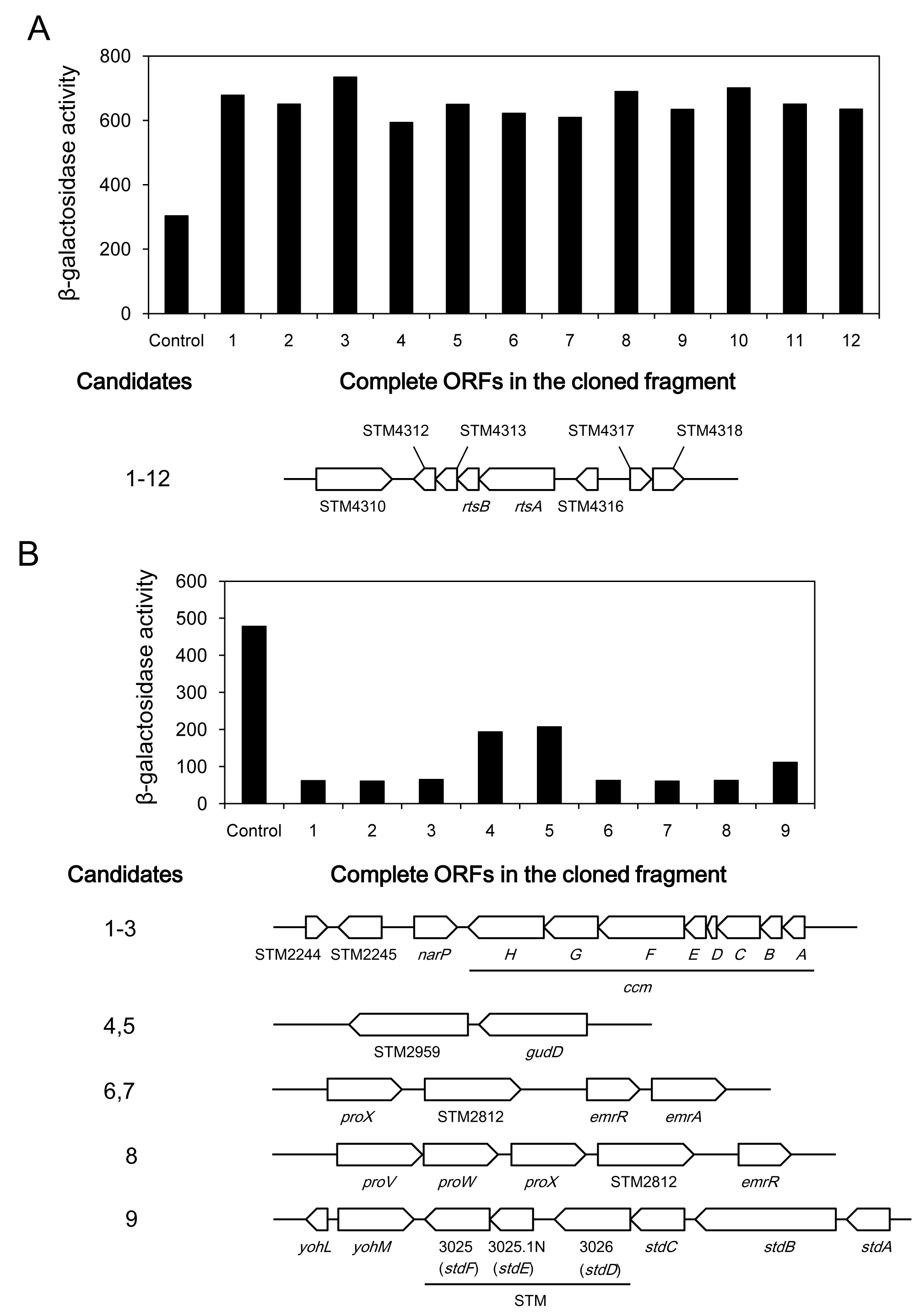

Supplement: Figure S3 — ß-galactosidase activity of the hilD::lac930 fusion in control strains (carrying pBR328), in candidates showing increased ß-galactosidase activity in a Dam− background (A) and in candidates showing reduced ß-galactosidase activity in a Dam+ background (B). Diagrams representing the fragments harbored by the plasmids present in the candidates are also shown. (TIF) [file pone.0030499.s003.tif]

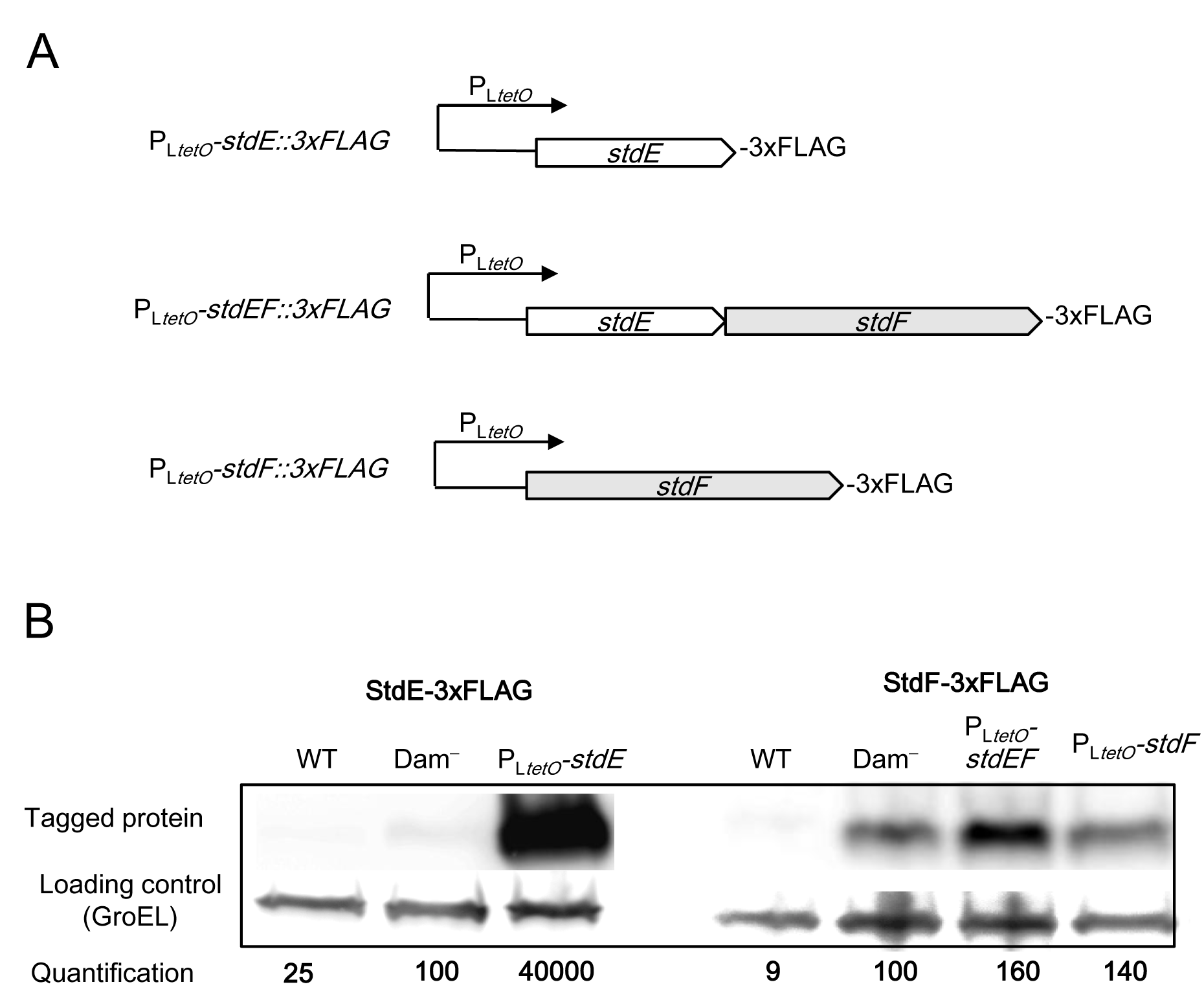

Supplement: Figure S4 — Production of StdE and StdF in strains carrying PLtetO -stdEF and PLtetO -stdF constructions A. Diagrams of strain construction and StdE and StdF tagging. B. Levels of StdE-3xFLAG and StdF-3xFLAG in protein extracts from the wild type, Dam−, PLtetO -stdEF, and PLtetO -stdF strains. 3xFLAG-tagged proteins were detected by Western blotting using a commercial anti-FLAG antibody. GroEL was used as loading control. For quantification, the ratio tagged protein/GroEL was relativized to 100 in the Dam− background. (TIF) [file pone.0030499.s004.tif]

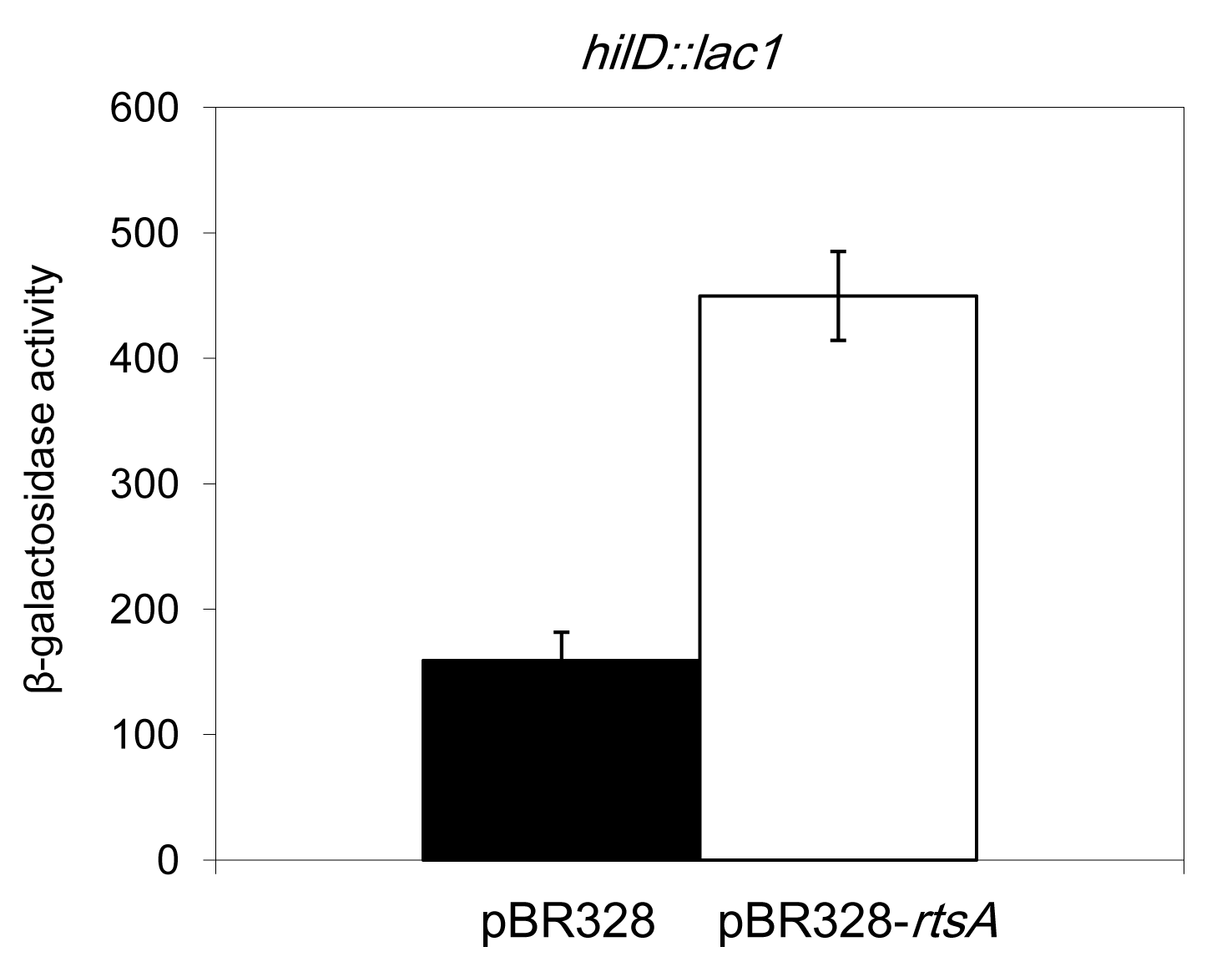

Supplement: Figure S5 — ß-galactosidase activity of the hilD::lac1 fusion in a strain carrying pBR328 (black histogram), and in a strain carrying a pBR328 derivative that contains the rtsA gene (white histogram). The differences observed are statistically significant (P<0.005). (TIF) [file pone.0030499.s005.tif]
