# Supplementary material for: Crosstalk between Virulence Loci: Regulation of Salmonella enterica Pathogenicity Island 1 (SPI-1) by Products of the std Fimbrial Operon
Source: PLoS One. 2012 Jan 23;7(1):e30499. doi: 10.1371/journal.pone.0030499 (PMC3264584; doi:10.1371/journal.pone.0030499)
Supplement: Table S1 — Strain list. (DOC) [file pone.0030499.s006.doc]

**Table S1. Strain list**

| Strain designation | Genotype or description | Reference or source |
| --- | --- | --- |
| ATCC 14028 | Wild type | ATCC |
| SV5264 | Δ*dam-231* | [39] |
| SV5284 | Φ(*hilA’-lacZ*^+^)(Hyb) | [39] |
| SV5297 | Φ(*invF’-lacZ*^+^)(Hyb) | [39] |
| SV5298 | Δ*dam-231* Φ(*invF’-lacZ*^+^)(Hyb) | [39] |
| SV5382 | Φ(*sipB’-lacZ*^+^)(Hyb) | [39] |
| SV5383 | Δ*dam-231* Φ(*sipB’-lacZ*^+^)(Hyb) | [39] |
| SV5457 | *invF*::3xFLAG | [39] |
| SV5456 | *hilA*::3xFLAG | [39] |
| SV5459 | *sipB*::3xFLAG | This study |
| SV6062 | *∆SPI-1* | F. Baisón |
| SV6410 | Φ(*hilD-lacZ1*) | This study |
| SV6413 | Φ(*hilD-lacZ930*) | This study |
| SV6473 | Δ*std* Φ(*invF’-lacZ*^+^)(Hyb) | This study |
| SV6474 | Δ*std* Δ*dam-231* Φ(*invF’-lacZ*^+^)(Hyb) | This study |
| SV6475 | Δ*std* Φ(*sipB’-lacZ*^+^)(Hyb) | This study |
| SV6476 | Δ*std* Δ*dam-231* Φ(*sipB’-lacZ*^+^)(Hyb) | This study |
| SV6477 | Δ*stdA* Φ(*invF’-lacZ*^+^)(Hyb) | This study |
| SV6478 | Δ*stdA* Δ*dam-231* Φ(*invF’-lacZ*^+^)(Hyb) | This study |
| SV6479 | Δ*stdB* Φ(*invF’-lacZ*^+^)(Hyb) | This study |
| SV6480 | Δ*stdB* Δ*dam-231* Φ(*invF’-lacZ*^+^)(Hyb) | This study |
| SV6481 | Δ*stdC* Φ(*invF’-lacZ*^+^)(Hyb) | This study |
| SV6482 | Δ*stdC* Δ*dam-231* Φ(*invF’-lacZ*^+^)(Hyb) | This study |
| SV6483 | Δ*stdD* Φ(*invF’-lacZ*^+^)(Hyb) | This study |
| SV6484 | Δ*stdD* Δ*dam-231* Φ(*invF’-lacZ*^+^)(Hyb) | This study |
| SV6485 | Δ*stdE* Φ(*invF’-lacZ*^+^)(Hyb) | This study |
| SV6486 | Δ*stdE* Δ*dam-231* Φ(*invF’-lacZ*^+^)(Hyb) | This study |
| SV6487 | Δ*stdF* Φ(*invF’-lacZ*^+^)(Hyb) | This study |
| SV6488 | Δ*stdF* Δ*dam-231* Φ(*invF’-lacZ*^+^)(Hyb) | This study |
| SV6501 | *stdF*::3xFLAG | This study |
| SV6502 | Δ*dam-231 stdF*::3xFLAG | This study |
| SV6503 | P_L_*_tetO_*-*stdEF* | This study |
| SV6504 | P_L_*_tetO_*-*stdF* | This study |
| SV6506 | P_L_*_tetO_*-*stdEF* Δ*stdE* | This study |
| SV6508 | P_L_*_tetO_*-*stdEF* Δ*stdF* | This study |
| SV6509 | P_L_*_tetO_*-*stdE*::3xFLAG | This study |
| SV6510 | P_L_*_tetO_*-*stdEF*::3xFLAG | This study |
| SV6511 | P_L_*_tetO_*-*stdEF* Φ(*invF’-lacZ*^+^)(Hyb) | This study |
| SV6512 | P_L_*_tetO_*-*stdEF* Φ(*sipB’-lacZ*^+^)(Hyb) | This study |
| SV6513 | P_L_*_tetO_*-*stdEF* Φ(*hilA’-lacZ*^+^)(Hyb) | This study |
| SV6515 | P_L_*_tetO_*-*stdEF* Φ(*hilD-lacZ1*) | This study |
| SV6517 | P_L_*_tetO_*-*stdF* Φ(*invF’-lacZ*^+^)(Hyb) | This study |
| SV6518 | P_L_*_tetO_*-*stdF* Φ(*sipB’-lacZ*^+^)(Hyb) | This study |
| SV6519 | P_L_*_tetO_*-*stdF* Φ(*hilA’-lacZ*^+^)(Hyb) | This study |
| SV6521 | P_L_*_tetO_*-*stdF* Φ(*hilD-lacZ1*) | This study |
| SV6524 | Φ(*hilD-lacZ1*)/pBR328 | This study |
| SV6525 | Φ(*hilD-lacZ1*)/pBR328-*rtsA* | This study |
| SV6530 | Φ(*stdD’-lacZ*^+^)(Hyb) | This study |
| SV6531 | Δ*dam-231* Φ(*stdD’-lacZ*^+^)(Hyb) | This study |
| SV6532 | Φ(*stdE’-lacZ*^+^)(Hyb) | This study |
| SV6533 | Δ*dam-231* Φ(*stdE’-lacZ*^+^)(Hyb) | This study |
| SV6534 | Φ(*stdF’-lacZ*^+^)(Hyb) | This study |
| SV6535 | Δ*dam-231* Φ(*stdF’-lacZ*^+^)(Hyb) | This study |
| SV6634 | P_L_*_tetO_*-*stdEF* Δ*stdEF* | This study |
| SV6635 | P_L_*_tetO_*-*stdF* Δ*stdF* | This study |
| SV6662 | *stdD*::3xFLAG | This study |
| SV6663 | Δ*dam-231 stdD*::3xFLAG | This study |
| SV6664 | P_L_*_tetO_*-*stdF*::3xFLAG | This study |
| SV6748 | *stdE*::3xFLAG | This study |
| SV6749 | Δ*dam-231 stdE*::3xFLAG | This study |
| SV6750 | P_L_*_tetO_*-*stdEF* Δ*stdE* Φ(*hilA’-lacZ*^+^)(Hyb) | This study |
| SV6751 | P_L_*_tetO_*-*stdEF* Δ*stdF* Φ(*hilA’-lacZ*^+^)(Hyb) | This study |
| SV6752 | P_L_*_tetO_*-*stdEF* Δ*stdEF* Φ(*hilA’-lacZ*^+^)(Hyb) | This study |
| SV6753 | P_L_*_tetO_*-*stdF* Δ*stdF* Φ(*hilA’-lacZ*^+^)(Hyb) | This study |
| SV6754 | P_L_*_tetO_*-*stdEF* Δ*stdE* Φ(*sipB’-lacZ*^+^)(Hyb) | This study |
| SV6755 | P_L_*_tetO_*-*stdEF* Δ*stdF* Φ(*sipB’-lacZ*^+^)(Hyb) | This study |
| SV6756 | P_L_*_tetO_*-*stdEF* Δ*stdEF* Φ(*sipB’-lacZ*^+^)(Hyb) | This study |
| SV6757 | P_L_*_tetO_*-*stdF* Δ*stdF* Φ(*sipB’-lacZ*^+^)(Hyb) | This study |
| SV6758 | P_L_*_tetO_*-*stdEF* Δ*stdE* Φ(*invF’-lacZ*^+^)(Hyb) | This study |
| SV6759 | P_L_*_tetO_*-*stdEF* Δ*stdF* Φ(*invF’-lacZ*^+^)(Hyb) | This study |
| SV6760 | P_L_*_tetO_*-*stdEF* Δ*stdEF* Φ(*invF’-lacZ*^+^)(Hyb) | This study |
| SV6761 | P_L_*_tetO_*-*stdF* Δ*stdF* Φ(*invF’-lacZ*^+^)(Hyb) | This study |
| SV6762 | P_L_*_tetO_*-*stdEF hilA*::3xFLAG | This study |
| SV6763 | P_L_*_tetO_*-*stdEF* Δ*stdE hilA*::3xFLAG | This study |
| SV6764 | P_L_*_tetO_*-*stdEF* Δ*stdF hilA*::3xFLAG | This study |
| SV6765 | P_L_*_tetO_*-*stdEF* Δ*stdEF hilA*::3xFLAG | This study |
| SV6766 | P_L_*_tetO_*-*stdF hilA*::3xFLAG | This study |
| SV6767 | P_L_*_tetO_*-*stdF* Δ*stdF hilA*::3xFLAG | This study |
| SV6768 | P_L_*_tetO_*-*stdEF sipB*::3xFLAG | This study |
| SV6769 | P_L_*_tetO_*-*stdEF* Δ*stdE sipB*::3xFLAG | This study |
| SV6770 | P_L_*_tetO_*-*stdEF* Δ*stdF sipB*::3xFLAG | This study |
| SV6771 | P_L_*_tetO_*-*stdEF* Δ*stdEF sipB*::3xFLAG | This study |
| SV6772 | P_L_*_tetO_*-*stdF sipB*::3xFLAG | This study |
| SV6773 | P_L_*_tetO_*-*stdF* Δ*stdF sipB*::3xFLAG | This study |
| SV6774 | P_L_*_tetO_*-*stdEF invF*::3xFLAG | This study |
| SV6775 | P_L_*_tetO_*-*stdEF* Δ*stdE invF*::3xFLAG | This study |
| SV6776 | P_L_*_tetO_*-*stdEF* Δ*stdF invF*::3xFLAG | This study |
| SV6777 | P_L_*_tetO_*-*stdEF* Δ*stdEF invF*::3xFLAG | This study |
| SV6778 | P_L_*_tetO_*-*stdF invF*::3xFLAG | This study |
| SV6779 | P_L_*_tetO_*-*stdF* Δ*stdF invF*::3xFLAG | This study |
| SV6780 | P_L_*_tetO_*-*stdEF* Δ*stdEF* Φ(*hilD-lacZ1*) | This study |
| SV6781 | P_L_*_tetO_*-*stdF* Δ*stdF* Φ(*hilD-lacZ1*) | This study |
| SV6788 | Δ*dam-231* Φ(*hilD-lacZ930*) | This study |
| SV6901 | P_L_*_tetO_*-*stdEF* Δ*stdEF trg*::Mu*d*J | This study |
| TR5878 | *galE496* r(LT2)^–^ m(LT2)^–^ r(S)^+^ *ilv-542 metA22 trpB2* Fels2^–^ *fliA66 strA120* *xyl-404 metE551 hspL56 hspS29* | J. R. Roth |
