# Supplementary material for: Crosstalk between Virulence Loci: Regulation of Salmonella enterica Pathogenicity Island 1 (SPI-1) by Products of the std Fimbrial Operon
Source: PLoS One. 2012 Jan 23;7(1):e30499. doi: 10.1371/journal.pone.0030499 (PMC3264584; doi:10.1371/journal.pone.0030499)
Supplement: Table S2 — Oligonucleotides used in this study (5′→3′). (DOC) [file pone.0030499.s007.doc]

**Table S2.** Oligonucleotides used in this study (5'→3')

| **Oligonucleotide** | **Sequence** |
| --- | --- |
| stdDUP | acgcaggggcgacatcatgacagaatggatttttaatctgattccggggatccgtcgacc |
| stdDDO | gattagttataggtaacagtaacgggtattgcagcagaaagtgtaggctggagctgcttc |
| stdD-E1 | tgcagatgaatcgctacacc |
| stdD-E1 | ttccccgataactcagtcag |
| stdEUP | ccagttatggagaggttttatgtgccctgataatacacacattccggggatccgtcgacc |
| stdEDO | ttaccgacccggcgttttgataccagcggcggtccggcttgtgtaggctggagctgcttc |
| stdE-E1 | tgctgcaatacccgttactg |
| stdE-E2 | caggctgcctgtatgcg |
| stdFUP | ggtccggagatttatgccgggctgcaactgtgaaaccgcaattccggggatccgtcgacc |
| stdFDO | tgtcagtgtttctggatagggtcgccggaggcgggttattgtgtaggctggagctgcttc |
| stdF-E1 | ggtcggtaatggtgacagg |
| stdF-E2 | gaaaggccatacattcagcg |
| stdD-3xFLAGUP | aacgttttctgctgcaatacccgttactgttacctataacgactacaaagaccatgacgg |
| stdD-3xFLAGDO | actcagtcagttcttttatttttattatctgcccccccgacatatgaatatcctccttag |
| stdF-3xFLAGUP | ttgtaaatcactgcagcgaaccccgtttatcgctccgcaagactacaaagaccatgacgg |
| stdF-3xFLAGDO | cggcgtgtcagtgtttctggatagggtcgccggaccgccccatatgaatatcctccttag |
| stdE-3xFLAGUP | gaagatcctgaaaaagaaggaggatgaggatgacggaacagactacaaagaccatgacgg |
| PLtetO-std UP | tacattaaaaagtatttctttgatgattattcttaaattaaggcttacccgtcttactgtc |
| PLtetO-stdEF-DO | ttcagggcacataaaacctctccataactgggtaaatgatgtgctcagtatctctatcactgatag |
| PLtetO-stdF-DO | cccgcatttctgttactgcacagccggttccacagttcatgtgctcagtatctctatcactgatag |
| stdA-FOR | atagccctgacagatgccg |
| stdB-REV | ggcctgcgacttcaggac |
| stdB-FOR | ctacctgacaggtctcagc |
| stdC-REV | gggtccggtcaacattgac |
| stdC-FOR | tgcagatgaatcgctacacc |
| stdD-REV | cctagctcaaccgcatacac |
| stdD-FOR | ctattacaggacgtgtcacc |
| stdE-REV | catcatggttggtctgtccg |
| stdE-FOR | ggtcggtaatggtgacagg |
| stdF-REV | gtttccgacgtaattgctgc |
| stdAUP | taaaattcttttcactggtaccatcaccaactcaccctgtattccggggatccgtcgacc |
| stdADO | ctgtcgttatttaccgcgtgaaatcacaggtatttcaggggtgtaggctggagctgcttc |
| stdAUP2 | taaaattcttttcactggtaccatcaccaactcaccctgtcatatgaatatcctccttag |
| stdA-E1 | ggaaagttcaggtgcttcg |
| stdA-E2 | gctttcgtgttgtcgtcc |
| stdBUP | gcgccatgccatgaaaattacccggcttgccattctgattattccggggatccgtcgacc |
| stdBDO | tttagacctgctctgtgacagggatatttttatctgcagggtgtaggctggagctgcttc |
| stdB-E1 | acgacagggagaagccg |
| stdB-E2 | cgcatccatgataatacgg |
| stdCUP | cacagagcaggtctaaaggagaaaacaggtgaaaaacagcattccggggatccgtcgacc |
| stdCDO | aatccattctgtcatgatgtcgcccctgcgtccttaaacggtgtaggctggagctgcttc |
| stdC-E1 | cggaaacggtgacactcag |
| stdC-E2 | tgaccggaagtgactgcac |
| **hilDriboprobeUP** | atggaaaatgtaacctttgtaag |
| **hilDriboprobeDO** | gtttttttaatacgactcactatagggaggtatatcgaaatccatgtggc |
| rnpBriboprobeUP | gaagaagtgaaactgaccgataagc |
| rnpBriboprobeDO | taatacgactcactataggccgaagctgaccagacagtcg |
| pBR328-Fw | actgtccgaccgctttgg |
| pBR328-Rv | gccagcaaccgcacctg |
| lacZ | gaccattttcaatccgca |
| hilDUP930 | aactacgccatcgacattcataaaaatggcgaaccattaacatatgaatatcctccttag |
| hilDUP1 | agagcatttacaactcagattttttcagtaggataccagtcatatgaatatcctccttag |
| hilDDO2 | gcaaatagttctcagagggaacggatgatgtataaatatggtgtaggctggagctgcttc |
| hilD-E1 | agaccattgccaacacacgc |
| hilD-E2’ | atcatcctcaggctggctcc |
